# Intensity modulated radiotherapy (IMRT) in patients with carcinomas of the paranasal sinuses: clinical benefit for complex shaped target volumes

**DOI:** 10.1186/1748-717X-1-23

**Published:** 2006-07-21

**Authors:** Stephanie E Combs, Stephan Konkel, Daniela Schulz-Ertner, Marc W Münter, Jürgen Debus, Peter E Huber, Christoph Thilmann

**Affiliations:** 1 University of Heidelberg, Department of Radiation Oncology, Im Neuenheimer Feld 400, 69120 Heidelberg, Germany; 2 German Cancer Center (dkfz), Clinical Cooperation Unit Radiation Oncology, Im Neuenheimer Feld 280, 69120 Heidelberg, Germany

## Abstract

**Introduction:**

The aim of the study was to evaluate the clinical outcome of intensity modulated radiotherapy (IMRT) in 46 patients with paranasal sinus tumors with special respect to treatment-related toxicity.

**Patients and methods:**

We treated 46 patients with histologically proven tumors of the paranasal sinuses with IMRT. Histological classification included squamous cell carcinoma in 6, adenocarcinoma in 8, adenoidcystic carcinoma in 20 and melanoma in 8 patients, respectively.

Six patients had been treated with RT during initial therapy after primary diagnosis, and IMRT was performed for the treatment of tumor progression as re-irradiation.

**Results:**

Overall survival rates were 96% at 1 year, 90% at 3 years.

Calculated from the initiation of IMRT as primary radiotherapy, survival rates at 1 and 3 years were 95% and 80%.

In six patients IMRT was performed as re-irradiation, and survival rate calculated from re-irradiation was 63% at 1 year.

Local control rates were 85% at 1, 81% at 2 and 49% at 3 years after primary RT and 50% at 1 year after re-irradiation.

Distant metastases-free survival in patients treated with IMRT as primary RT was 83% after 1 and 64% after 3 years. For patients treated as primary irradiation with IMRT, the distant control rate was 83% at 1 year and 0% at 2 years.

No severe radiation-induced side-effects could be observed.

**Conclusion:**

IMRT for tumors of the paranasal sinuses is associated with very good tumor control rates. Treatment-related acute and long-term toxicity can be minimized as compared to historical results with conventional RT.

## Introduction

Tumors of the paranasal sinuses (PNS) and nasal cavity are relatively rare, accounting for about 3–5% of all head and neck tumors; they are commonly associated with a poor prognosis [1,2]. Their incidence amounts to about 0.5% of all malignant diseases, and they show a wide variety of histologic subtypes [3]. The presence of air filled spaces permits silent growth of these tumors, and symptoms often occur only after the tumor has reached a considerable volume. Therefore, the majority of patients presents with advanced tumors, often extending into the skull base in close vicinity to sensitive risk structures such as optic nerves, chiasm, eyes and brain stem [4-6].

The primary treatment of choice is an aggressive surgical approach, followed by postoperative radiotherapy (RT) [7,8]. Due to the complexity of the anatomy and the proximity of these neoplasms to critical normal tissue structures radical surgery is often not possible [9-14]; furthermore, RT is associated with a high risk of treatment-related toxicity [15-17]. In the past, chronic toxicity to the optic system was of major concern, with RT-induced blindness rates of up to 37% [17-19]. Furthermore, underdosage in regions of risk were a major concern with conventional RT techniques.

With modern high-precision RT-techniques such as Intensity Modulated Radiotherapy (IMRT), it is possible to increase the dose to defined target volume while reducing the dose to limiting organs at risk (OAR) to preserve organ function and subsequently quality of life. Furthermore, it is possible to improve dose conformality to the target volume with this technique as compared to conventional conformal RT techniques.

Previous clinical results published from our institution have shown that IMRT can be applied safely and effectively in patients with tumors of the PNS [20]. However, these results were confined to a small number of patients only with a short follow-up time.

The present study retrospectively evaluates the results of IMRT in 46 patients with carcinomas of the PNS, with special respect to treatment related acute and chronic toxicity.

## Patients and methods

### Patients' characteristics

Between January 1999 and October 2005, we treated 46 patients with histologically proven tumors of the PNS with IMRT. All patients were followed regularly after treatment.

Patients' characteristics are summarized in table 1. Histological classification included squamous cell carcinoma (SCC) in 6, adenocarcinoma (AC) in 12, adenoidcystic carcinoma (ACC) in 20 and melanoma in 8 patients.

**Table 1:** Patient and disease characteristics of 46 patients with paranasal sinus carcinomas treated with IMRT

	**N (%)**
**Gender**	
female	18 (39%)
male	28 (61%)
**Histology**	
Adenocarcinoma	8 (17.4%)
Squamous Cell Carcinoma	6 (13%)
Adenoid-cystic Carcinoma	20 (43.4%)
Melanoma	8 (17.4%)
other	4 (8.8%)
**Primary tumor site**	
Maxillary sinus	22 (47.8%)
Ethmoidal sinus	4 (8.7%)
Sphenoid sinus	4 (8.7%)
Nasal cavity	16 (34.8%)
**Tumor stage**	
T1	2 (4%)
T2	3 (7%)
T3	11 (24%)
T4	30 (65%)
**Nodal stage**	
N0	34 (74%)
N1	6 (13%)
N2	1 (2%)
Nx	5 (11%)

Patients with benign tumors such as inverted papilloma and with palate or skin primary tumors with secondary invasion of the sinuses and the nose were excluded from the analysis. Accordingly, pediatric sarcomas and esthesioneuroblastomas invading the PNS were not included.

The tumor site was determined from the epicenter of the disease, as determined at the time of diagnosis or, more rarely, from an analysis of the clinical, radiologic or operative data. The sub site of origin was the maxillary sinus in 22 patients, the sphenoid sinus in 4, the ethmoidal sinus in 4 patients, and the nasal cavity in 16 patients, respectively. All patients were staged according to the 2002 TNM classification system [21]. Five patients presented with T1/T2 tumors, 11 with T3 and 30 patients with T4 tumors, respectively.

Six patients (13%) presented with intracranial invasion of the tumor; in 11 patients (24%) the orbit was infiltrated.

Six out of 46 patients (13%) had been treated with RT during initial therapy after primary diagnosis, and IMRT was performed as re-irradiation for the treatment of tumor progression.

### Treatment planning

All patients were treated with IMRT using the step-and-shoot approach [22]. For treatment planning, patients were fixed in an individually manufactured precision head mask made of Scotch cast^® ^(3 M, St.Paul, Minneapolis, MN), allowing a treatment setup accuracy of 1–2 mm [23]. If treatment of the lymph nodes was required patients were additionally positioned with an individually fixed vacuum pillow in order to immobilize the neck and thorax. With this immobilization system attached to the stereotactic base frame, we performed contrast-enhanced CT- and MRI-images under stereotactic conditions, with a slice thickness of 3 mm. We scanned the whole treatment region with a superior and inferior margin of at least 3 cm.

After stereotactic image fusion based on the localizer-derived coordinate system [24,25], all critical structures including the optic nerves, chiasm, brainstem and eyes as well as the target volumes were defined on each slice of the three-dimensional data cube. The Gross Tumor Volume (GTV) was defined as the macroscopic tumor visible on CT- and MRI-scans. For the clinical target volume (CTV) a generous margin was added according to the typical pathways for microscopic spread and the anatomical relationship of adjacent structures. Generally, the CTV consisted of the resection cavity, all PNS completely or partially invaded, adding a safety margin of 3–5 mm. No elective RT of the cervical lymph nodes was performed. If treatment of the local lymph nodes was necessary, they were defined as a part of the CTV.

Inverse treatment-planning was performed using the KonRad software developed at the German Cancer Research Center (dkfz), which is connected to the 3D planning program VIRTUOS to calculate and visualize the 3D dose distribution. With the KonRad planning programme, dose constraints and penalties for the target volumes as well as the organs at risk must be defined prior to starting the optimization process. The couch, gantry and collimator angles as well as the number of beams and intensity levels can be varied. Treatment planning has been described in detail previously [20,26-28].

Treatment was delivered by a Siemens accelerator (Primus, Siemens, Erlangen, Germany) with 6 or 15 MV photons using an integrated motorized multileaf collimator (MLC) for the step-and-shoot technique automatically delivering the sequences.

The total doses were prescribed to the median of the target volume, meaning 50% of the target volume receives 100% of the dose. The median target volume was chosen for dose prescription since is represents the majority of the defined target volume.

For dose prescription we adhered to the tolerance doses of each organ at risk; dose constraints were set at 27 Gy for the parotid, 54 Gy for the optic nerves, chiasm and brain stem and 45 Gy for the spinal cord. Dose prescription was performed, with respect to these tolerance doses, after plan calculation and analyses of the dose volume histogram (DVH). A summary of the DHV data is provided in table 2.

**Table 2:** Summary of the DVH-data

***Characteristics***	***Mean***	***SEM***	***Median***	***Range***
**Boost(CTV)**				
D_med _(Gy)	66	12,4	66	36–78
V_>30% _(%)	99,97	0,08	100	99,7–100
V_<90% _(%)	8,18	18,5	4,3	0,4–100
Volume (cm^3^)	177,8	102,93	153,7	26,5–402,4
**PTV**				
D_med _(Gy)	56,8	12,4	60	23,6–67,5
V_>30% _(%)	99,91	0,37	100	98–100
V_<90% _(%)	25,8	20,15	21,3	1,7–70,4
Volume (cm^3^)	475,6	380,8	342,2	34–1524
**Right Optic Nerve**				
D_med _(Gy)	35,5	11,47	37,9	9,3–62,5
V_>30% _(%)	93,98	13,8	100	35,3–100
V_<90% _(%)	90,93	23,3	100	13,4–100
Volume (cm^3^)	1,3	0,42	1,4	0,5–2,4
**Left Optic Nerve**				
D_med _(Gy)	34,39	11,52	37,4	13,2–60,5
V_>30% _(%)	89,9	20,01	100	10–100
V_<90% _(%)	92,2	21,75	100	14,4–100
Volume (cm^3^)	1,2	0,48	1,2	0,5–2,2
**Chiasm**				
D_med _(Gy)	24,79	8,4	25,3	9,4–41,4
V_>30% _(%)	72,88	31,56	83,05	0–100
V_<90% _(%)	97,52	25,66	100	0,96–100
Volume (cm^3^)	1,5	0,6	1,56	0,2–2,9
**Brain Stem**				
D_med _(Gy)	24,9	9,95	25,2	9,9–63,2
V_>30% _(%)	66,5	24,95	70,7	0,6–100
V_<90% _(%)	99,9	0,19	100	99,1–100
Volume (cm^3^)	27,5	5,35	12,8	12,8–35,2

For patients treated with IMRT as adjuvant RT after surgery, the median total dose applied was 64 Gy in a median fractionation of 2 Gy (range 1.8 – 2.2 Gy). A median total dose of 54 Gy was prescribed to the CTV, and a median dose of 64 Gy was prescribed to the GTV as a boost. For irradiation of the lymph nodes, a median dose of 54 Gy was applied.

In 6 patients IMRT was performed as re-irradiation with a median dose of 46 Gy in a median fractionation of 2 Gy (range 1,8 Gy – 2 Gy). Prior to IMRT, a median total dose of 62 Gy had been applied using conventional RT.

### Follow-up

All patients were followed prospectively after RT. A thorough clinical assessment including contrast-enhanced MRI- or CT-scans as well as an ultrasound were scheduled 6 weeks after completion of RT, then in 3-months intervals for the first year. Thereafter, follow-up visits were scheduled every 6 months or as needed clinically. Additionally, patients were followed by otorhinolaryngologist as well as ophthalmologist on a regular basis.

Acute and late therapy-related side effects were scored according to the Common Toxicity Criteria (CTC) version 3.0 of the U.S. National Institutes of Health.

### Statistics

Local tumor control, distant-metastases-free survival, survival from RT as well as overall survival were determined using the Kaplan-Meier-Method [29], calculated from the initiation of RT. Overall survival was defined as the survival time calculated from the primary diagnosis of the PNS tumor. Survival from IMRT was calculated as the survival time starting from the initiation of IMRT. All analyses were performed using the Statistica software (StatSoft 6.0, Germany).

## Results

### Treatment in general

**Figure 1:**
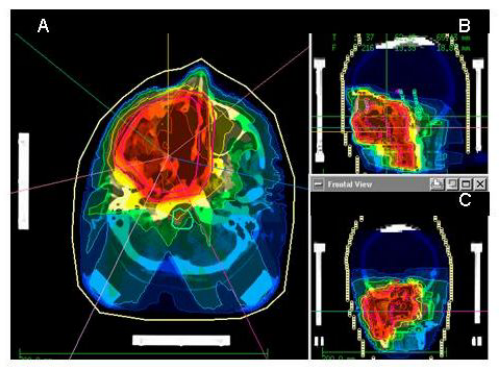
Typical IMRT treatment plan of a patient treated for paranasal sinus carcinoma: transversal view (A), sagital view (B) and coronar view (C).

A typical IMRT treatment plan is depicted in Fig. 1. The main goal was optimal sparing of the optic structures as well as the brain stem, without compromising the dose conformality to the CTV and PTV. A summary of the DVH-data can be found in table 2.

### Overall survival

The median follow-up time was 16 months (range 3–40 months).

Nine patients died of tumor progression during follow-up. Thirty-seven patients were alive at the time point of analysis. Overall survival rates were 96% at 1 year. Tumor stage, histology, primary site of origin and the presence of orbital and intracranial invasion did not influence overall survival; however, numbers of patients in each subdivision might be too small to reach statistical significance.

No treatment-related deaths occurred.

### Survival after IMRT

**Figure 2:**
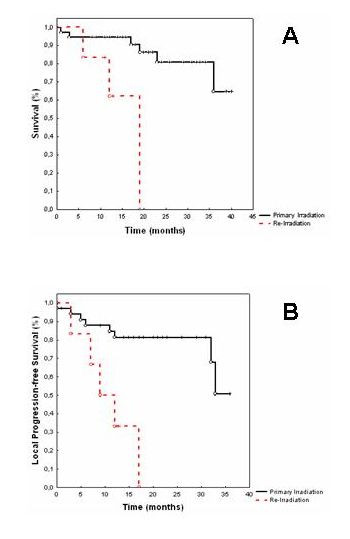
Survival calculated from the initiation of IMRT (A). Local progression-free survival calculated from the initiation of IMRT (B).

Calculated from the initiation of IMRT as adjuvant radiotherapy, survival rates at 1 year was 95% (Fig. 2A).

In six patients IMRT was performed as re-irradiation, and survival rate calculated from re-irradiation was 63% at 1 year (Fig. 2A).

Primary site of origin, presence of orbital or intracranial invasion, histological subtype and tumor stage did not influence survival after primary irradiation or re-irradiation significantly, most probably due to the small number of patients in each subgroup.

### Local control

In patients were IMRT was performed as adjuvant RT, local control rates were 85% at 1 and 81% at 2 years, respectively (Fig. 2B). Local control after IMRT performed as re-irradiation was 50% at 1 year. We could not identify any significant prognostic factors for local tumor control including histology, tumor stage, intracranial and orbital extension of the tumor and primary site of origin. Again, this could be due to the relatively small number of patients within each subclassification.

### Distant tumor control

**Figure 3:**
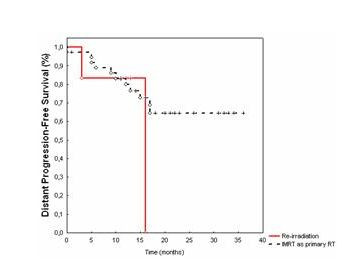
Distant metastases-free survival calculated from the initiation of IMRT performed as primary irradiation (black curve) and as re-irradiation (red curve).

Distant metastases-free survival in patients treated with IMRT as adjuvant RT was 83% after 1 year (Fig. 3).

For patients treated as re-irradiation with IMRT, the distant control rate was 83% at 1 year and 0% at 2 years.

No prognostic factors for distant metastases-free survival could be found (Table 3).

### Acute and chronic toxicity

Minor acute side effects of RT included focal alopecia, nausea/vomiting and fatigue.

The development of severe radiation-induced side-effects could be prevented in our study group. Mucositis developed in most patients, however, Grade 2 and 3 mucositis developed in 8 and 2 patients only. No Grade IV reactions to the mucosa could be observed. Skin erythema developed in 28 patients, in 19 as CTC grade I and 9 as CTC grade II. No grade III and IV erythema could be observed.

Radiation-induced conjunctivits developed in 12 patients as CTC grade I and in 1 as CTC grade II. No radiation-induced visual deficits could be observed. All patients were seen and the side effects documented by an ophthalmologist.

During follow-up, 19 patients presented with mild xerostomia (CTC grade I) and 2 patients with CTC grade II. No severe (CTC grade III and IV) xerostomia could be observed.

No other severe side effects could be observed.

## Discussion

The management of PNS cancers remains a major challenge in oncology. A major problem in patients with carcinomas of the PNS is that most tumors are highly advanced at the time point of diagnosis. Commonly, early symptoms differ little from ordinary nasal complaints, and their temporary regression by antibiotics misleads both the patients and the physicians [30-32]. Thereafter, if symptoms recur or should more alarming symptoms such as visual defects, cranial nerve deficits or a visible mass in the head-and-neck area develop, tumor stage is commonly T3 or T4, with outcome tending to be less favourable [33]. Most probably, the presence of large air spaces and the fast growth pattern of the most common histologies allow the fast and asymptomatic expansion of PNS carcinomas. Tumor volumes, even after complete surgical resection of the tumor, are therefore relatively large, in the majority of cases affecting all sinuses.

Until now, there is substantial controversy as to which treatment can be considered the "standard treatment approach": surgery, definitive radiotherapy or a combined multimodality approach including surgical resection followed by postoperative surgery. For single-modality therapies, outcome is generally poor. Amendola et al. reported on 39 patients treated with curative intent with resection or definitive RT and found no statistically significant differences in survival at 3 and 5 years, with a 5 year survival rate of 31% and 35% for resection and RT, respectively [34]. A number of reports have demonstrated some improvement in outcome with combined modality therapy. St. Pierre and Baker reported on 61 patients treated with surgical resection alone, definitive RT or combined treatment, showing a clear benefit for patients receiving combined surgery and RT [35]. Paulino and colleagues could show in a group of 48 patients that local control and disease-specific survival rates at 5 years were significantly increased in the group receiving surgery and RT as compared to RT alone, with an overall survival rate of 52% and 0%, respectively [36]. Blanco et al. demonstrated that disease-free survival increased slightly with a multimodality treatment approach, however, overall survival was unaltered [37]. In our group all patients were treated with IMRT, in 6 patients IMRT was performed as re-irradiation for tumor progression. Overall survival was 96% at 1 year. Calculated from the initiation of IMRT, survival rates for the group treated with primary RT was 95% at 1 year. This rate is relatively high as compared to data reported in the literature. This might be due to the high number of patients with ACC included into this analysis, showing a smaller rate of local and distant progression than other histologies such as SCC, SC or Melanoma. However, within this analysis, neither histology, nor other common prognostic factors did significantly influence outcome; most probably this is due to the relatively small number of patients in each subgroup.

In the past, the main concern in the radiotherapeutic treatment of PNS tumors was treatment-related toxicity. The close vicinity of sensitive organs at risk such as the eyes, optic nerves, chiasm and brain stem makes it difficult to apply a high and effective dose to the target volume while sparing healthy tissue using conventional RT techniques. A number of groups have reported unilateral and bilateral blindness rates after conventional RT of PNS tumors up to 60% and 10% of the patients, respectively [38-40]. In other series, the rate for radiation-induced blindness ranges from 15% to 40% [17-19,19,41]. However, Karim et al. showed that by applying a shrinking technique for the target volume and thus preventing irradiation of the whole orbit in patients with orbital invasion did not influence outcome negatively, while the ocular structures were excluded from the high-dose regions. However, RT-induced blindness was rare (4%) [42,43].

The introduction of IMRT now allows application of high doses to complex target volumes, while the surrounding OARs can be spared and toxicity may be reduced. Over the last years, IMRT has been implemented widely into the clinical routine. However, most publications to date have focused on treatment planning techniques and theoretical plan comparisons of IMRT plans as compared to conformal RT plans [5,40,44-47]. For tumors of the PNS the potential benefits of IMRT are obvious due to the anatomical site: the target volumes to be treated are in very close vicinity to sensitive normal tissues and organs at risk (OAR), especially the eyes, optic nerves, chiasm, brain stem and spinal cord. With conventional RT techniques, the dose application to the target volumes is limited by the tolerated doses of the OAR in order to avoid high rates of treatment-related toxicity.

Until now, only a small number of groups have reported their results of IMRT in patients with carcinomas of the PNS. Duthoy et al. published their results of IMRT in 39 patients with PNS cancers [48]. The median dose delivered in that study was 70 Gy, and the actuarial overall survival rates were 68% at 2 and 59% at 4 years, respectively. The actuarial local control rates were 73% and 68% at 2 and 4 years, respectively. However, acute toxicity was mild, and no patient developed Grade or 4 ocular toxicity. Two patients developed decreased vision after RT, however, no RT-induced blindness was observed. Our results are in good accordance with these data.

The development of severe radiation-induced side-effects could be prevented in our study group as well. Mucositis developed in most patients, however, Grade 2 and 3 mucositis developed in 8 and 2 patients only. No Grade IV reactions to the mucosa could be observed. A significant number of patients developed ocular side effects, however, no Grade 3 and 4 reactions occurred, especially, no RT-induced blindness. However, follow-up time still remains relatively short.

The results of the present study therefore confirm the idea that IMRT can lead to equal local control and survival rates as compared to conventional or conformal RT in patients with carcinomas of the PNS. However, with IMRT, OARs in close vicinity to the target volume can be spared effectively. Thus, the risk of severe treatment related side effects especially to the optic system can be minimized.
